# Loss of Lipooligosaccharide Synthesis in *Acinetobacter baumannii* Produces Changes in Outer Membrane Vesicle Protein Content

**DOI:** 10.3390/ijms25179272

**Published:** 2024-08-27

**Authors:** Beatriz Cano-Castaño, Andrés Corral-Lugo, Eva Gato, María C. Terrón, Antonio J. Martín-Galiano, Javier Sotillo, Astrid Pérez, Michael J. McConnell

**Affiliations:** 1 Intrahospital Infections Laboratory, Instituto de Salud Carlos III (ISCIII), National Centre for Microbiology, 28220 Madrid, Spain or bcano65@alumno.unes.es (B.C.-C.); eva.gato@isciii.es (E.G.); astrid.perez@isciii.es (A.P.); 2 Escuela internacional de Doctorado, Ciencias de la Salud, Universidad Nacional de Educación a Distancia (UNED), 28015 Madrid, Spain; 3 Protein Synthesis Quality Control, Institute of Genetics and Development of Rennes, 35000 Rennes Cedex, France; andres.corrallugo@gmail.com; 4 Electron Microscopy Unit, Instituto de Salud Carlos III (ISCIII), 28220 Madrid, Spain; mcterron@isciii.es; 5 Core Scientific and Technical Units, Instituto de Salud Carlos III (ISCIII), 28220 Madrid, Spain; mgaliano@isciii.es; 6 Parasitology Reference and Research Laboratory, Instituto de Salud Carlos III (ISCIII), National Centre for Microbiology, 28220 Madrid, Spain; javier.sotillo@isciii.es; 7 Department of Biological Sciences, University of Notre Dame, Notre Dame, IN 46556, USA; 8 Eck Institute for Global Health, University of Notre Dame, Notre Dame, IN 46556, USA

**Keywords:** outer membrane vesicles, proteomic, *Acinetobacter baumannii*, lipooligosaccharide, vaccine, OMVs

## Abstract

Outer membrane vesicles (OMVs) are nanostructures derived from the outer membrane of Gram-negative bacteria. We previously demonstrated that vaccination with endotoxin-free OMVs isolated from an *Acinetobacter baumannii* strain lacking lipooligosaccharide (LOS) biosynthesis, due to a mutation in *lpxD*, provides full protection in a murine sepsis model. The present study characterizes the protein content of highly-purified OMVs isolated from LOS-replete and LOS-deficient strains. Four purification methods were evaluated to obtain highly purified OMV preparations: ultracentrifugation, size exclusion chromatography (SEC), ultracentrifugation followed by SEC, and Optiprep™. OMVs from each method were characterized using nanoparticle tracking analysis and electron microscopy. OMVs from LOS-deficient and LOS-replete strains purified using the Optiprep™ method were subjected to LC-MS/MS analysis to determine protein content. Significant differences in protein composition between OMVs from LOS-deficient and LOS-replete strains were found. Computational analyses using Bepipred 3.0 and SEMA 2.0 indicated that the lack of LOS led to the overexpression of immunogenic proteins found in LOS-containing OMVs and the presence of immune-stimulating proteins absent in LOS-replete OMVs. These findings have important implications for developing OMV-based vaccines against *A. baumannii*, using both LOS-containing and LOS-free OMVs preparations.

## 1. Introduction

Outer membrane vesicles (OMVs) are a type of extracellular vesicle derived from the outer membranes of Gram-negative bacteria [1]. OMVs are one of multiple different types of extracellular vesicles that have been described in bacteria. These include vesicles that originate from the inner membrane and can contain cytosolic material (outer–inner membrane vesicles) [2] and vesicles that are formed via explosive cell lysis and concomitant self-annealing of shattered membrane fragments [3]. As structures that originate from the bacterial outer membrane, the antigen repertoire of OMVs reflects that of the membrane from which they are derived [4,5]. Their small size, typically 40–400 nm in diameter [6], facilitates their uptake by antigen-presenting cells. Moreover, bacterial membrane antigens located in OMVs are presented to lymphocytes in their native conformation [7,8]. For these reasons, OMVs are being intensively studied for use as vaccines to prevent bacterial infections [9]. Previous studies have shown that vaccination with these entities can induce protective immunity to multiple Gram-negative and Gram-positive bacterial pathogens, including *Escherichia coli* [10], *Bordetella pertussis* [11], and *Staphylococcus aureus* [12]. In addition, vaccines containing OMVs have been approved for human use, for example, Bexsero^®^ in Europe, or Vamengoc-BC^®^ in Cuba, both for the prevention of *Neisseria meningitidis* infections [13,14].

*Acinetobacter baumannii* is a Gram-negative pathogen that causes serious pulmonary, bloodstream, and urinary tract infections, most commonly in critically ill patients [15]. Currently, the treatment of multidrug-resistant *A. baumannii* infections is complicated by the limited number of clinically available antibiotics that retain activity against this pathogen. In addition, due to the dissemination of multidrug-resistant strains worldwide [16], the World Health Organization has classified this microorganism as a priority pathogen for which the development of new treatments is urgently needed [17]. The ability of *A*. *baumannii* to acquire resistance to new antibiotics highlights the importance of developing alternative therapeutic options. The use of vaccines to reduce the morbidity and mortality due to infections caused by multidrug-resistant pathogens such as *A. baumannii* has been recognized as a potential alternative to relying solely on antimicrobial therapy [18]. Active and passive immunization strategies for *A. baumannii* infections have previously been explored in experimental animal models [19]. Among these approaches, *A. baumannii* OMVs have been studied as potential vaccines antigens [20,21]. Vaccination with *A. baumannii* OMVs protects mice against infection with clinical isolates in both pneumonia and sepsis models [22], and mice vaccinated with OMVs have lower bacterial loads in organs and fluids compared to those unimmunized mice [22,23,24]. In a separate study, recombinant OMVs from *E. coli* expressing outer membrane protein 22 from *A. baumannii* demonstrated significant protection in a mouse model of infection [25].

As structures originating from the outer membrane, OMVs derived from Gram-negative bacteria also contain high levels of lipopolysaccharide (LPS), which is highly reactogenic [8,26]. LPS binds to host toll-like-receptor 4, which triggers the release of diverse mediators of inflammation, such as TNF-α and IL1-β. Due to such adverse effects, limits have been established regarding the amount of LPS that can be present in pharmaceutical products [27]. Although LPS is a component of the outer membrane that is essential for viability in most Gram-negative species [28], *A. baumannii* tolerates the absence of LPS biosynthesis [29]. Notably, the *A. baumannii* LPS lacks the O-antigen, and is, thus, termed lipooligosaccharide (LOS) [30].

In a previous study, we demonstrated that an *lpxD*–deficient *A. baumannii* strain completely deficient in LOS produced OMVs with undetectable endotoxin levels [21]. Immunization with these LOS-free OMVs significantly reduces post-infection tissue bacterial load and provides full protection in a lethal murine sepsis model [21]. These data suggest that LOS-free OMVs derived from *A. baumannii* is a promising and safe vaccine candidate, since it may lack the toxicity associated with the presence of LOS found in OMVs derived from wild-type *A. baumannii* strains. However, loss of LOS biosynthesis in *A. baumannii* has been shown to alter the composition of the bacterial outer membrane, which may affect the efficacy of LOS-free OMVs-based vaccines [31]. In the present study, we gain insights into the antigenic proteome of OMVs derived from LOS-replete (ATCC 19606) and LOS-deficient *A. baumannii* (IB010) strains by comparing four different methodologies for obtaining highly purified OMV preparations and performing quantitative proteomics. This study is, therefore, the first to quantitatively characterize differences between native OMVs and OMVs lacking LOS in *A. baumannii*, and highlights how loss of LOS biosynthesis affects the protein composition of *A. baumannii* OMVs.

## 2. Results

### 2.1. Comparison of Methods for Purifying A. baumannii OMVs

**Figure 1:**
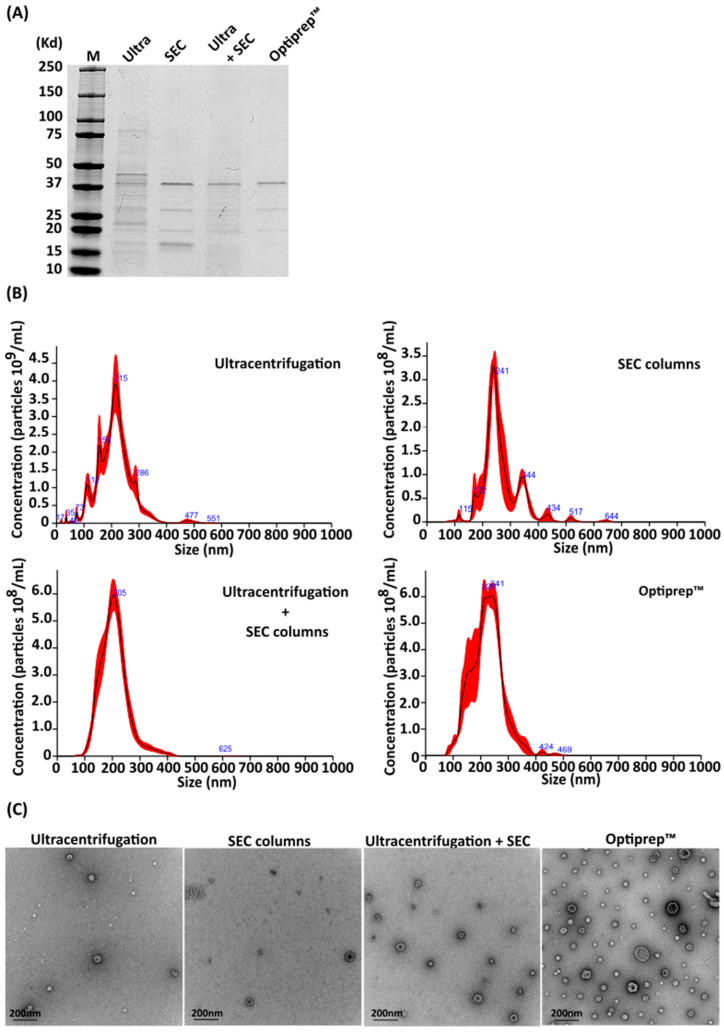
Comparison of four methodologies for OMVs isolation. (**A**) Coomassie staining of selected fractions of OMVs derived from ATCC 19606 isolated with different methodologies. (**B**) Nanoparticle tracking analysis histograms of OMVs isolated from *A. baumannii* ATCC 19606 with different techniques. (**C**) Transmission electron micrograph of OMVs purified from A. baumannii ATCC 19606. The scale bar represents 200 nm.

Multiple methods are available for the purification of bacterial OMVs. Here, we evaluated four different methodologies for isolation of *A. baumannii* OMVs, namely, ultracentrifugation alone, Size Exclusion Chromatography (SEC) columns, ultracentrifugation plus SEC columns, and Optiprep^TM^ gradients. The protein profiles of *A. baumannii* ATCC 19606 OMVs purified using these four techniques were compared by sodium dodecyl sulphate polyacrylamide gel electrophoresis (SDS-PAGE) and Coomassie staining. As shown in Figure 1A, OMVs purified using ultracentrifugation alone exhibited a smear of multiple proteins that were not found in the OMVs purified using the other three methods. indicating contamination by non-OMV polypeptides. Conversely, OMVs obtained using the other three methods demonstrated similar protein profiles.

OMVs purified using each of the four methods were characterized using nanoparticle tracking analysis (NTA) in order to assess OMV size distribution and concentration (Figure 1B). A concentration of 3.54 × 10^11^ ± 2.91 × 10^10^ particles per mL was obtained with double ultracentrifugation, 2.59 × 10^10^ ± 3.12 × 10^9^ particles/mL using SEC columns, 6.18 × 10^10^ ± 4.49 × 10^9^ particles/mL with ultracentrifugation and size-exclusion chromatography, and 7.85 × 10^10^ ± 5.68 × 10^9^ particles/mL with the Optiprep™ protocol. As shown in Figure 1B, OMVs purified using either ultracentrifugation or SEC columns alone resulted in the isolation of particles of different sizes, whereas ultracentrifugation in combination with SEC column and Optiprep™ gradients produced OMV preparations with homogeneous particles.

The purity of OMVs obtained using each method was expressed as particles per µg of protein, as described previously [32]. Optiprep™ purification resulted in the highest ratio, at 1.96 × 10^10^ particles per µg of protein. Combining ultracentrifugation and size-exclusion chromatography also demonstrated efficacy, yielding a ratio of 3.90 × 10^9^ particles/µg of protein. Using only size-exclusion chromatography led to lower purity compared to the combined approach (1.20 × 10^9^ particles/µg of protein). Ultracentrifugation resulted in the lowest purity, producing OMVs with a ratio of 5.76 × 10^8^ particles/µg of protein.

All samples were qualitatively analyzed by transmission electron microscopy (TEM) in order to assess OMV morphology. While all purification methodologies resulted in bilayer particles with a size consistent with that previously reported for Gram-negative OMVs [33,34,35], OMVs obtained using the Optiprep™ protocol were qualitatively more homogeneous in morphology compared to OMVs obtained using the other three methods, and demonstrated less non-OMV material in the preparations (Figure 1C).

### 2.2. Comparison of ATCC 19606 and IB010 OMVs

**Figure 2:**
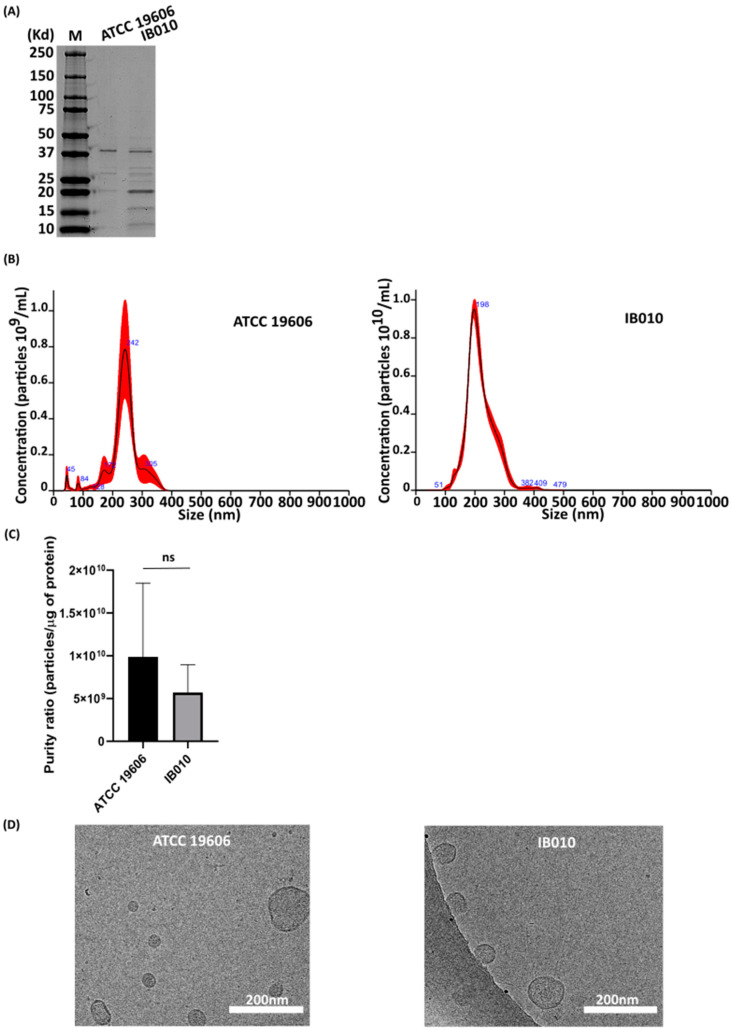
Characterization and comparison of OMVs derived from *A. baumannii* ATCC 19606 and its LOS-deficient derivative isolated by Optiprep™ methodology. (**A**) Coomassie staining of ATCC 19606 and IB010 OMVs. (**B**) NTA histograms of OMVs isolated via Optiprep™ centrifugation density gradient from *A. baumannii* ATCC19606 and IB010. (**C**) Purity of OMVs isolated with Optiprep™ from ATCC 19606 and IB010. The left bar represents particles/µg of protein obtained from ATCC 19606 (9.87 × 10^10^) and the bar on the right represents particles/µg of protein obtained from IB010 (5.70 × 10^9^); ns, non-statistically significant. (**D**) CryoEM images of OMVs from *A. baumannii* strains. Scale bar represents 200 nm.

Since the Optiprep™ purification protocol yielded the most homogenous population of OMV particles and with the highest purity, this technique was selected for purifying OMVs for further analyses. Three biological replicates of OMV preparations were isolated from LOS-replete ATCC 19606 and its isogenic LOS-deficient derivative IB010. After SDS-PAGE and Coomassie staining, the purified OMVs from these strains indicated similar, albeit not identical, protein profiles (Figure 2A).

NTA revealed that IB010 OMV preparations resulted in a consistently higher number of particles/mL in comparison to the parental strain, with 75.3 ± 1.32 × 10^10^ particles/mL and 5.42 ± 1.46 × 10^10^ particles/mL, respectively. As shown in Figure 2B, NTA indicated that highly homogeneous particle sizes were obtained for OMVs from both the ATCC 19606 strain and IB010. Notably, the major population of OMVs obtained from the ATCC 19606 strain were 242 nm in size, whereas those from the IB010 strain were 198 nm. The mean purity ratio of OMVs from triplicate samples from each strain were 9.87 × 10^9^ particles/µg of protein for ATCC 19606 and 5.70 × 10^9^ particles/µg of protein for IB010 (Figure 2C).

When purified OMVs were examined using cryoelectron microscopy (cryoEM), both strains produced OMVs with an appreciable bilayer membrane with sizes consistent with previously reported sizes of OMVs from Gram-negative bacteria (Figure 2D) [2,34,36].

### 2.3. Proteomic Characterization of ATCC 19606 and IB010 OMVs

**Figure 3:**
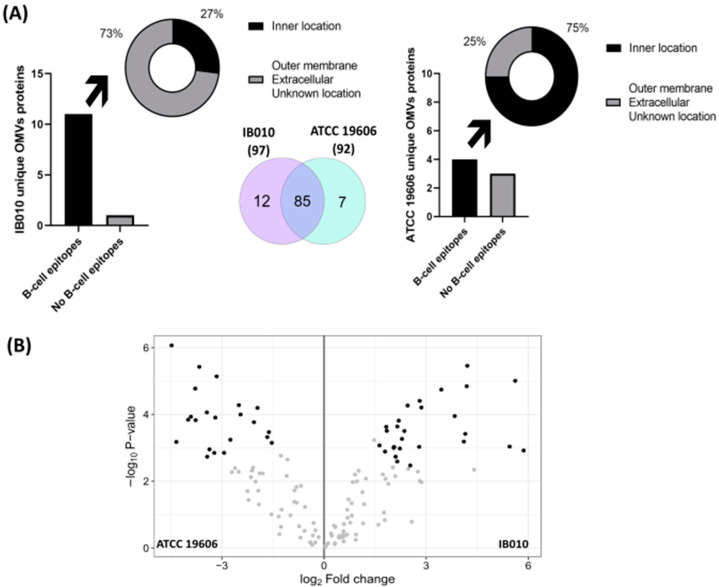
Proteomic characterization of *A. baumannii* OMVs. (**A**) Venn diagram illustrating protein distribution between OMVs isolated from *A. baumannii* strains ATCC 19606 and IB010. The diagram indicates that OMVs from ATCC 19606 possess seven unique proteins, of which 4 had B-cell epitopes and 25% of it could be recognized by b-cells for its location. OMVs from IB010 possess 12 unique proteins, of which 11 had B-cell epitopes and 73% of it could be recognized by B-cells for its location. The OMVs from both strains share 85 proteins. (**B**) Volcano plot showing differential expression between OMVs proteins of IB010 and ATCC 19606. The adjusted *p*-value threshold is set at 0.01 (colored dots). Proteins with log2 fold change greater than 1.5 or less than −1.5 are considered significantly overexpressed or underexpressed, respectively.

Three biological replicates of purified OMVs from both strains were used to assess protein content using liquid chromatography tandem mass spectrometry (LC-MS/MS). A total of 92 proteins were identified in ATCC 19606 OMVs, whereas 97 proteins were identified in IB010 OMVs (Figure 3A). Of these proteins, seven were detected only in ATCC 19606 OMVs (Table 1) and twelve were detected only in IB010 OMVs (Table 2). Of the 85 proteins that were found in both ATCC 19606 OMVs and IB010 OMVs, 16 proteins were at least 1.5-fold overexpressed in the ATCC 19606 strain compared to IB010, and 17 proteins demonstrated at least 1.5-fold reduced expression in the ATCC 19606 strain compared to IB010 (Figure 3B, Table 3, Supplementary Table S3). These results indicate that the loss of LOS biosynthesis results in the production of OMVs with altered protein composition. This is consistent with a previous study indicating that loss of LOS biosynthesis functions in *A. baumannii* results in altered protein composition of the bacterial outer membrane [31].

### 2.4. Computational Characterization of Differentially Expressed Proteins

Since OMVs from both wild-type and LOS-deficient *A. baumannii* have been characterized as potential vaccine candidates in preclinical models, we performed an immunoinformatic analysis on differentially expressed proteins. Bepipred 3.0 was used to predict linear B-cell epitopes in proteins that were over- and underexpressed in OMVs from the LOS-deficient strain (Supplementary Table S1). Of the 16 overexpressed proteins, all of them had at least one predicted linear epitope that could be recognized by B-cells. Two proteins had two epitopes (Accession codes: D0CBP8; D0C6J4; https://www.uniprot.org (accessed on 24 October 2023). Three proteins, including an OmpA family protein, and a lipoprotein (D0C9R5; D0CEN2; D0C5H7) had three B-cell epitopes. Another lipoprotein, a hemolysin, and a DJ-1/PfpI family protein contained four epitopes (D0C608; D0C8L9; D0CDU8). Two proteins with unknown location had five epitopes (D0CDW1; D0C5N9), and one uncharacterized protein had six epitopes (D0CBW9). Seven epitopes were present in the toluene tolerance protein Ttg2D (D0CCX7). In contrast, 7 underexpressed proteins out of 17 had no predicted linear immunogenic regions. Four proteins, including Rhombotarget A and 30S ribosomal protein S3, had one epitope (D0CBK4; D0CD03; D0C6N7; D0C5L6). An M23 family peptidase and a lytic murein transglycosylase B had two epitopes (D0CBZ2; D0CC83). Only three proteins contained three and four epitopes, including a Tat pathway signal sequence domain protein (D0CDN5), and a Type VI secretion system effector, Hcp1 family (D0C8P3; D0CD52).

The subcellular location of OMV-associated proteins was assigned by PSORTb 3.0.3. A total of 21 proteins out of 104 are from the outer membrane, 11 come from the cytoplasmic membrane, 16 are periplasmic, 5 come from the cytoplasm, 4 are extracellular, and 4 proteins come from multiples sites. The remaining proteins have unknown locations.

Overexpressed proteins in IB010 OMVs identified in outer membrane protein by PRED-TMBB (http://bioinformatics.biol.uoa.gr/PRED-TMBB/ (accessed on 4 October 2023) with a VaxyJen 2.0 immunogenicity score over 0.5 and β-barrel structure predicted were an OmpA family protein, OmpH like protein, and a Signal peptide (D0C9R5; D0C6H4; D0C5N9). As can be seen in Supplementary Figure S1, these proteins were predicted to have rich discontinuous epitopes areas predicted by SEMA 2.0, with a coverage of epitopic residues per total of amino acids of 36%, 66%, and 64%, respectively. Underexpressed proteins analyzed with the same criteria were an M23 family peptidase and a Tat pathway signal domain protein (D0CBZ2; D0CDN5), with 49% and 43% of epitopic residues/total amino acids (Supplementary Table S1).

Linear epitopes in proteins found only in IB010 or ATCC 19606 OMVs were also predicted with Bepipred 3.0. Of the proteins detected only in IB010 OMVs, only one of them had no linear epitopes (D0CDY8), although it was predicted to have β-barrel structure, to be antigenic by Vaxijen 2.0 and to have 25% of epitope/total residues by SEMA 2.0. SEMA 2.0 predicted discontinuous epitopes in the CarO protein, containing 44% epitope/total residues (Supplementary Table S2). Three of the seven proteins that were not present in IB010 OMVs had no predicted linear B-cell epitopes, and only one protein that had unknown location was an uncharacterized protein with discontinuous epitopes predicted by SEMA 2.0 (D0CCD3).

## 3. Discussion

In this study, we identified changes in OMVs protein composition occurring due to loss of LOS biosynthesis in *A. baumannii*. This revealed clues for the development of safe OMV-based vaccines for *A. baumannii* using LOS-free preparations. OMVs have previously been shown to induce protective immunity when used as vaccine antigens [14].

As a common technical bottleneck in OMV approaches, in the present study, we optimized a method for obtaining very-high-purity preparations of *A. baumannii* wild-type and LOS-free OMVs. Previous studies employed only ultracentrifugation to purify OMVs from *A. baumannii* cultures. OMVs can be isolated through different techniques, such as ultracentrifugation (including differential centrifugation), density gradient centrifugation (such as sucrose gradient and Optiprep™), filtration, size-exclusion chromatography, or other less commonly used methodologies [37]. The International Society for Extracellular Vesicles (ISEV) indicates that there is no single optimal separation method, so it is recommended to screen several methods to identify the most appropriate one depending on downstream applications, taking into account whether more quantity or purity of OMVs is needed [38]. The results presented here indicate that, of the four methods tested, the Optiprep™ protocol results in homogenous OMVs of high purity. In agreement with our results, Steć et al. showed a significantly greater purity in extracellular vesicles isolated from the plant pathogen *Pectobacterium zantedeschiae* using this methodology over two other techniques, including ultracentrifugation [39].

Their acellular nature, small size, and ability to carry antigenic proteins in their native conformation makes OMVs an attractive option for vaccine development. In our previous studies, the efficacy of a vaccine based on *A. baumannii* OMVs completely free of LOS was similar to that observed using control OMVs with LOS [21]. Importantly, a previous study showed that *A. baumannii* deficient in LOS biosynthesis has altered membrane composition compared to wild-type strains containing LOS [31]. The fact that OMVs are globally originated from the bacterial membrane raises the possibility that the altered antigen content of OMVs derived from LOS-deficient strains may also negate its applicability for vaccine development. We have demonstrated that although the absence of LOS does alter protein composition of OMVs compared to wild-type OMVs, they retain almost 85% of the proteins, with moderate abundance variation. This concordance includes most antigenic proteins detected in OMVs. For example, OMVs from both strains harbor immunogenic proteins such as Omp38 and the outer protein assembly factor BamA, which have been extensively studied as a vaccine candidates in *A. baumannii* [40]. OmpA, also named as omp38, has been described as a very important component of OMVs in this species due to its role in OMVs formation [41,42]. BamA and BamD have been found in OMVs from the clinical *A. baumannii* strain DU202 [43]. The TolB protein, which has been predicted to be a promising vaccine antigen [44], has been also found in OMVs proteomic studies using the ATCC 19606 strain [45]. In agreement with the results obtained by Hassan et al. [46], a lipoprotein (HMPREF0010_02142) and a peptidyl-prolyl cis-trans isomerase (HMPREF0010_03292) found in both OMVs could be potentially used for vaccination.

Furthermore, all proteins that were overexpressed in the LOS-deficient strain have at least one predicted linear epitope that could be recognized by B-cells, in comparison to the underexpressed proteins, where more than 40% had no predicted B-cell linear epitope. Moreover, both an OmpA family protein and an OmpH-like protein had predicted discontinuous epitopes and were more abundant in IB010 OMVs. Contrary to our results, OmpH-like proteins were underexpressed in whole cells of LOS-deficient strains of *A. baumannii* [47]. Henry et al. made a genetic study in an isogenic LOS-deficient strain of ATCC 19606 that exhibited reduced expression of genes encoding a type VI secretion system in comparison to the wild type [48], in agreement with our findings in IB010 OMVs. On the other hand, the absence of LOS induced the expression of immunogenic proteins such as CarO, a protein that has been studied as a potential vaccine antigen in *A. baumannii* [49]. In contrast, the LOS deficiency did not appear to affect humoral antigenicity since most of the unique proteins in ATCC 19606 OMVs did not have predicted B-cell epitopes or are located in cellular compartments not accessible to antibodies in the *A. baumannii* cells.

## 4. Materials and Methods

### 4.1. Purification of A. baumannii OMVs

The *A. baumannii* reference strain ATCC 19606 and its isogenic LOS-deficient derivative IB010, which contains a 462 base pair deletion in the *lpxD* gene [50], were used in this study. In order to compare different OMV purification techniques, a 10 mL starter culture containing the indicated strains in Mueller Hinton II broth (MHB) was grown at 37 °C for eight hours, and then diluted 1/100 in MHB and incubated for an additional 16 h at 37 °C with shaking at 180 rpm. OMVs were isolated from 200 mL of culture unless otherwise indicated. Cells were pelleted by centrifugation (Sorvall LYNX 4000; fixed angle rotor Fiberlite™ F14-6 x 250y. Thermo Fisher Scientific, Schwerte, Germany) at 10,000× *g* for 30 min at 4 °C, and the supernatant was filtered through a 0.22 μm vacuum filter Steritop-GP polyethersulfone membrane (Millipore, Burlington, MA, USA) before concentrating the sample using a solvent-resistant stirred cell (Millipore, Molsheim, FR, USA) with 10 kDa ultrafiltration discs (Millipore, Jaffrey, NH, USA). Supernatants were further concentrated using 10 kDa centrifuge filtration units (Amicon^®^ Ultra-15 Millipore, Tullagreen, CO, USA) and filtered through a 0.22 μm syringe filter (Corning, Sigma-Aldrich, Darmstadt, Germany) before being used in one of the four purification methods described below.

For purification by ultracentrifugation, concentrated supernatants were centrifuged at 100,000× *g* for 6 h at 4 °C (SW40.Ti rotor, Beckman, Munich, Germany). The resulting pellet was resuspended in 10 mL of PBS and then sterilized with a 0.2 µm syringe before centrifugation at 100,000× *g* for 6 h at 4 °C. The resulting pellet was resuspended in 200 µL of sterile PBS and filtered through a 0.2 µm syringe filter. Protein concentration was measured using Bradford assay (Bio-Rad, Madrid, Spain), and particle concentration was obtained through nanoparticle tracking analysis.

For purification by size-exclusion chromatography, SEC qEV isolation columns (qEV2/70 nm Legacy Column; Izon, Bonsai Lab, Madrid, Spain) were used to purify OMVs according to the manufacturer’s instructions. Concentrated and filtered supernatants were applied to the columns, and 20 fractions of 1 mL were collected in PBS. Each fraction was analyzed by immunoblot using an anti-*A. baumannii* omp22 antibody in order to detect fractions containing OMVs. Protein and particle concentrations, obtained as described above, were determined to assess the purity of each fraction. The purest fraction was then used to compare purification techniques.

For purification using ultracentrifugation followed by size-exclusion chromatography, concentrated and filtered supernatants were centrifuged at 100,000× *g* for 6 h, at 4 °C. The resulting pellet was resuspended in 2 mL of PBS and the sample was applied to SEC qEV isolation columns according to manufacturer’s instructions. Fractions were collected in PBS and analyzed as described above.

For purification using Optiprep™ gradients (Sigma-Aldrich, Darmstadt, Germany), a previously described protocol was employed [51], with the following modifications. Briefly, five sucrose concentrations were generated for gradient ultracentrifugation (50%, 40%, 20%, 10%, and 0%), centrifugation was carried out at 4 °C for 16 h and 30 min at 100,000× *g* and 16 fractions were collected. For proteomic analysis, Optiprep™ was eliminated from samples by diluting each 1 mL fraction in 5 mL of PBS and centrifuging at 150,000× *g* for 1 h and 30 min. The purest fractions containing OMVs were pooled together.

OMVs isolated using each of the four techniques were plated on Mueller Hinton II agar plates to confirm the absence of viable bacteria and stored at −80 °C until use.

For Coomassie staining, 500 ng of purified OMVs were subjected to 12% SDS-PAGE) and stained with Coomassie brilliant blue R-250 [52].

### 4.2. Nanoparticle Tracking Analysis (NTA)

Samples containing purified OMVs were diluted in PBS, and were analyzed by NTA using a NanoSight NS300 (Malvern, Madrid, Spain) with NTA 3.4 Build 3.4.003 software. During capture, screen gain was set to 8, and camera level to between 11 and 13. While processing results, detection threshold was set to between 2 and 4. Samples were assessed under controlled flow, using the NanoSight syringe pump and script control system. Videos of 30–60 s of duration were recorded, with a 10 s delay between recordings, generating replicate histograms (3 replicates per sample).

The purity of OMV preparations was determined by dividing the number of particles per mL obtained using NTA by the protein concentration measured for each methodology, as described previously [32].

### 4.3. Electron Microscopy and Cryoelectron Microscopy

OMVs were analyzed by transmission electron microscopy with negative staining. Five μL aliquots of the OMV samples were fixed for 5 min with 5 μL of 4% paraformaldehyde in PBS. OMVs were then adsorbed onto glow-discharged (Q15OT ES, Quorum Technologies, Lewes, UK) collodion carbon-coated grids (CF300-Cu, Electron Microscopy Sciences, Hatfield, PA, USA) for 5 min, washed two times with Milli-Q water, and negatively stained with 2% aqueous uranyl acetate for 1 min. The samples were then analyzed with an FEI Tecnai 12 electron microscope (Thermo Fisher, Schwerte, Germany) equipped with a LaB6 filament operated at 120 kV. Images were recorded with an FEI Ceta CCD camera (Thermo Fisher Scientific, Schwerte, Germany).

In addition, cryoelectron microscopy analysis of OMV was performed. Five μL aliquots of the OMV preparations were applied to Quantifoil Cu/Rh R2/2 300 mesh glow-discharged grids and vitrified using a Leica EM GP2 cryofixation unit (Leica, Wetzlar, Germany). Data were collected on a Talos cryoelectron microscope (Thermo Fisher Scientific, Schwerte, Germany) operated at 200 kV, and images were recorded with TFS Falcon 3 Direct Electron Detector in linear mode using TFS EPU 3 Automated Data Acquisition Software for Single Particle Analysis.

### 4.4. Mass Spectrometry Analysis (LC-MS/MS)

OMVs purified using the Optiprep™ protocol were used for LC-MS/MS analysis. For sample preparation, the µL of each sample solution corresponding to 1.5 µg P_t_ were brought up to 20 µL with 50 mM ABC.

In solution digestion: Cysteine residues were reduced by 2 mM DTT (DL-Dithiothreitol) in 50 mM ABC at 60 °C for 20 min. Sulfhydryl groups were alkylated with 5 mM IAM (iodoacetamide) in 50 mM ABC in the dark at r.t. for 30 min. IAM excess was neutralized with 10 mM DTT in 50 mM ABC, 30 min at r.t.

Samples were subjected to trypsin digestion with 100 ng of sequencing grade-modified trypsin (Promega, Madrid, Spain) in 50 mM ammonium bicarbonate at 37 °C overnight. The reaction was stopped with trifluoroacetic acid at a final concentration of 0.1%. Four μL of the digestion mixture were diluted to 20 μL with 0.1% formic acid and loaded onto an Evotip pure tip (EvoSep, Odense, Denmark) according to the manufacturer’s instructions. LC–MS/MS was performed with the EvoSep One system connected to a Tims TOF fleX mass spectrometer (Bruker, Madrid, Spain). Samples were eluted to an analytical column (PepSep C18 8 cm × 100 µm, 3 µm; Bruker, Madrid Spain) via the EvoSep One system and resolved using the 100 SPD chromatographic method defined by the manufacturer. The eluted peptides were ionized in a captive Spray with 1700 V at 200 °C and analyzed in a ddaPASEF mode. Per strain, three replicates were performed.

LC-MS/MS results were analyzed with Fragpipe (https://fragpipe.nesvilab.org/ (accessed on 4 October 2023) [53]. The workflow selected was LFQ-MBR, running MSFragger with a precursor mass tolerance of ±10 ppm and a fragment mass tolerance of 5 ppm. MSBooster was selected and an FDR filter of 0.05 was applied. IonQuant (http://ionquant.nesvilab.org/ (accessed on 4 October 2023) was used for protein quantification and database was downloaded from Uniprot (*A. baumannii* 19606; organism ID: 470). Raw data files were analyzed using FragPipe-Analyst (http://fragpipe-analyst.nesvilab.org/ (accessed on 4 October 2023) to obtain protein identifications and their respective label-free quantification values using in-house standard parameters. Statistical analysis was performed based on the combined_protein.tsv file. First, contaminant proteins were filtered out. In addition, proteins that were not identified/quantified consistently under the same condition were removed. The MaxLFQ intensity values were converted to log2 scale, samples were grouped by conditions, and missing values were imputed using the “Missing not At Random” (MNAR) method, which uses random draws from a left-shifted Gaussian distribution of 1.8 StDev (standard deviation) apart with a width of 0.3. Proteins with more than 1 missing value were discarded. Protein-wise linear models combined with empirical Bayes statistics were used for the differential expression analyses. The limma package from R Bioconductor 3.60.4 was used to generate a list of differentially expressed proteins for each pair-wise comparison. A cutoff of the adjusted *p*-value of 0.01 (Benjamini–Hochberg method) along with a |log2 fold change| of 1.5 was applied to determine significant differences in proteins.

### 4.5. Subcellular Localization and Epitope Prediction

Protein subcellular localizations were predicted using PSORTb 3.0.3 tool [54]. Bepipred 3.0 [55] was used to predict linear B-cell epitopes with a threshold of 0.15, with a minimum of seven continuous epitopic residues considered and SEMA 2.0 [56] to predict discontinuous epitopes using 0.51 as the cut-off value. Outer membrane proteins that had at least one linear B-cell epitope were subjected to VaxiJen 2.0 [57] antigenicity prediction and SEMA 2.0 to confirm discontinuous epitopes. Those proteins with unknown subcellular localization that had a B-cell epitope in addition to an immunogenicity score ≥ 0.5 were subjected to PRED-TMBB (http://bioinformatics.biol.uoa.gr/PRED-TMBB/ (accessed on 4 October 2023) [58] to determine if they contained beta-barrel motifs consistent with outer membrane proteins. SEMA 2.0 was also used on these proteins.

## 5. Conclusions

Collectively, our results validate the utilization of LOS-free OMVs from viable mutants as safe vaccines for *A. baumannii*. Such OMVs carry most protective relevant antigens, and in some cases even in higher amounts than wild-type counterparts. We envisage that this strategy has passed the proteomic filter and warrant experimental validation in animal models. Additionally, these results indicate significant differences in protein composition between OMVs produced by LOS-replete and LOS-deficient *A. baumannii* strains.

## Figures and Tables

**Table 1:** Unique proteins in OMVs derived from *A. baumannii* ATCC 19606.

Protein ID	Gene Name	Protein Description	Combined Total Peptides
D0CG85	*tuf*	Elongation factor Tu (Fragment)	5
D0CDQ1	*bpeB*	Efflux pump membrane transporter	4
D0C629	*HMPREF0010_00209*	Translocation and assembly module subunit TamA	3
D0CEK8	*atpF*	ATP synthase subunit b	3
D0C807	*gltI*	Glutamate-aspartate periplasmic-binding protein	2
D0CCD3	*HMPREF0010_02413*	Uncharacterized protein	6
D0C7Q4	*HMPREF0010_00784*	Uncharacterized protein	2

Outer membrane vesicles (OMVs). Combined total peptides: total number of peptides (stripped sequences) mapping to the selected protein, including shared peptides.

**Table 2:** Unique proteins in OMVs derived from *A. baumannii* LOS-deficient derived strain IB010.

Protein ID	Gene Name	Protein Description	Combined Total Peptides
D0C7A8	*HMPREF0010_00638*	Lipoprotein	3
D0CDQ8	*lolA*	Outer-membrane lipoprotein carrier protein	3
D0C9M0	*lolB*	Outer-membrane lipoprotein LolB	2
D0CC74	*rpsB*	30S ribosomal protein S2	2
D0CDB4	*HMPREF0010_02744*	DUF306 domain-containing protein	2
D0CDY8	*HMPREF0010_02968*	TonB-dependent siderophore receptor	2
D0CF71	*HMPREF0010_03537*	Carbapenem susceptibility porin CarO	2
D0CFX6	*HMPREF0010_03656*	Metallo-beta-lactamase domain protein	2
D0CBN6	*HMPREF0010_02166*	Uncharacterized protein	3
D0CF26	*HMPREF0010_03356*	Uncharacterized protein	3
D0C7G2	*HMPREF0010_00692*	Uncharacterized protein	2
D0CB14	*HMPREF0010_01944*	Uncharacterized protein	2

Outer membrane vesicles (OMVs). Combined total peptides: total number of peptides (stripped sequences) mapping to the selected protein, including shared peptides.

**Table 3:** Over- and underexpressed proteins in OMVs of IB010.

Protein ID	Gene Name	Protein Description	IB010 vs. ATCC 19606 log_2_ Fold Change	*p* Value
I. Overexpressed proteins				
D0C9R5	*HMPREF0010_01378*	OmpA family protein	4.19	<0.01
D0C907	*HMPREF0010_01565*	META domain protein (Fragment)	2.81	<0.01
D0CEN2	*HMPREF0010_03212*	Lipoprotein	2.8	<0.01
D0CCX7	*ttg2D*	Toluene tolerance protein Ttg2D	2.46	<0.01
D0C6H4	*HMPREF0010_00354*	Outer membrane protein	2.19	<0.01
D0C5N9	*HMPREF0010_00069*	Signal peptide protein	2.15	<0.01
D0CDW1	*HMPREF0010_02941*	Lipoprotein	2.15	<0.01
D0CDU8	*HMPREF0010_02928*	Lipoprotein	2.1	0.02
D0C6J4	*gdhB*	Quinoprotein glucose dehydrogenase B	2.06	<0.01
D0CBP8	*mucD*	Periplasmic serine endoprotease DegP-like	1.84	<0.01
D0C608	*HMPREF0010_00188*	DJ-1/PfpI family protein	1.83	<0.01
D0C8L9	*HMPREF0010_01099*	Hemolysin	1.79	0.01
D0CDA9	*HMPREF0010_02739*	Uncharacterized protein	5.62	<0.01
D0CBW9	*HMPREF0010_02249*	Uncharacterized protein	3.44	<0.01
D0CFX4	*HMPREF0010_03654*	Uncharacterized protein	2.86	<0.01
D0C5H7	*HMPREF0010_00007*	Uncharacterized protein	2.05	0.01
II. Underexpressed proteins				
D0CEW0	*ptk*	Tyrosine-protein kinase Ptk (Fragment)	−4.48	<0.01
D0CBK4	*HMPREF0010_02134*	Rhombotarget A	−3.99	<0.01
D0C5L7	*mrcB*	Penicillin-binding protein 1B	−3.78	<0.01
D0CBK5	*HMPREF0010_02135*	Gammaproteobacterial enzyme transmembrane domain protein	−3.36	0.01
D0CD03	*rpsC*	30S ribosomal protein S3	−3.22	<0.01
D0C9Z6	*mqo*	Probable malate:quinone oxidoreductase	−3.15	<0.01
D0C8P3	*HMPREF0010_01123*	Type VI secretion system effector, Hcp1 family	−2.93	0.01
D0CDE5	*gcd*	Quinoprotein glucose dehydrogenase	−2.45	<0.01
D0CEV8	*HMPREF0010_03288*	Polysaccharide biosynthesis/export protein	−2.06	<0.01
D0CC83	*mltB*	Lytic murein transglycosylase B	−1.67	<0.01
D0CBZ2	*HMPREF0010_02272*	Peptidase, M23 family	−1.62	<0.01
D0CDN5	*HMPREF0010_02865*	Tat pathway signal sequence domain protein	−1.53	<0.01
D0C6H8	*HMPREF0010_00358*	Uncharacterized protein	−3.66	<0.01
D0C6N7	*HMPREF0010_00417*	Uncharacterized protein	−3.44	<0.01
D0C985	*HMPREF0010_01198*	Uncharacterized protein	−2.75	<0.01
D0CD52	*HMPREF0010_02682*	Uncharacterized protein	−2.5	<0.01
D0C5L6	*HMPREF0010_00046*	Uncharacterized protein	−1.95	<0.01

## Data Availability

The data presented in this study are available on request from the corresponding author.

## Supplementary Materials

The following supporting information can be downloaded at: https://www.mdpi.com/article/10.3390/ijms25179272/s1.
