# Supplementary material for: Loss of Lipooligosaccharide Synthesis in Acinetobacter baumannii Produces Changes in Outer Membrane Vesicle Protein Content
Source: Int J Mol Sci. 2024 Aug 27;25(17):9272. doi: 10.3390/ijms25179272 (PMC11395390; doi:10.3390/ijms25179272)
Supplement: Supplementary file 1 [file ijms-25-09272-s001.zip › ijms-3176244-supplementary.pdf]

Supplementary material

Table S1. Computational assessment of differential expressed proteins

Protein ID	Protein Description	B-cell epitopes [a]	Predicted location [b]	VaxiJen score [c]	PRED-TMBB score [d]	Discontinuos epitopes [e]		
						Residues [f]	Total residues	Residues/total residues (%)
**I.**	**Overexpressed proteins in IB010 OMVs**							
D0CCX7	Toluene tolerance protein Ttg2D	7	Unknown	0.5459	2.986	69	189	37
D0C5N9	Signal peptide protein	5	Unknown	0.5027	2.929	59	167	35
D0C608	DJ-1/PfpI family protein	4	Periplasmic space					
D0C8L9	Hemolysin	4	Unknown	0.638	3.008	72	198	36
D0C9R5	OmpA family prot	3	Outer membrane	0.7523	2.834	72	217	33
D0CEN2	Lipoprotein	3	Unknown	0.75	3.003	73	108	68
D0CDW1	Lipoprotein	3	Unknown	0.5026	2.997	71	146	49
D0CDU8	Lipoprotein	3	Unknown	0.3641	2.980	71	202	38
D0C6J4	Quinoprotein glucose dehydrogenase B	2	Unknown	0.4484	0.2983	117	455	26
D0C6H4	Outer membrane protein	1	Unknown	0.6212	2.950	95	145	66
D0C907	META domain fragment	1	Unknown	0.6924	3.012	105	337	31

D0CBP8	Periplasmic serine endoprotease DegP-like	1	Periplasmic space					
D0CBW9	Uncharacterized protein	6	Unknown	0.6187	2.965	61	212	29
D0C5H7	Uncharacterized protein	3	Unknown	0.2152	3.011	58	150	39
D0CDA9	Uncharacterized protein	1	Periplasmic space					
D0CFX4	Uncharacterized protein	1	Unknown	0.6185	3.169	75	87	86
**II.**	**Under expressed proteins in IB010 OMVs**							
D0C8P3	Type VI secretion system effector, Hcp1 family	4	Extracellular	0.881	2.984	51	167	31
D0CDN5	Tat pathway signal sequence domain protein	3	Outer membrane	0.7889	2.870	213	435	49
D0CBZ2	Peptidase, M23 family	2	Outer membrane	0.6465	2.933	119	276	43
D0CC83	Lytic murein transglycosylase B	2	Cytoplasmic membrane					
D0CBK4	Rhombotarget A	1	Unknown	0.4863	2.959	212	598	35
D0CD03	30S ribosomal protein S3	1	Cytoplasm					
D0C9Z6	Probable malate:quinone oxidoreductase	0						
D0C5L7	Penicillin-binding protein 1B	0						

D0CBK5	Gammaproteobacterial enzyme transmembrane domain protein	0							
D0CEW0	Protein-tyrosine kinase ptk	0							
D0CDE5	Quinoprotein glucose dehydrogenase	0							
D0CEV8	Polysaccharide biosynthesis/export protein	0							
D0C6H8	Uncharacterized protein	2	Extracellular	0.6984	2.985	142	273	52	
D0C6N7	Uncharacterized protein	1	Extracellular	0.5434	2.979	161	476	34	
D0CD52	Uncharacterized protein	4	Unknown	0.6349	3	60	188	32	
D0C5L6	Uncharacterized protein	1	Unknown	0.4208	3.031	94	159	59	
D0C985	Uncharacterized protein	0							

a) B-cell epitopes predicted by Bepipred 3.0 with a threshold > 0.15; b) Subcellular location predicted with PSORTb; c) VaxiJen treshold > 0.5 d) β-barrel conformation threshold < 2.95; e) Discontinuous epitopes predicted with SEMA with a treshold > 0.51; f) 7 or more consecutive amino acid residues predicted as discontinuous epitope regions by SEMA

Table S2. Computational assessment of unique proteins expressed in OMVs derived from *A. baumannii* ATCC19606 or its LOS-deficient derived strain IB010

Protein ID	Protein description	B-cell epitopes [a]	PSORTb location [b]	VaxiJen score [c]	PRED-TMBB score [d]	SEMA score [e]		
						Epitopic residues [f]	Total residues	Residues/Total residues (%)
**I.**	**Unique proteins in 19606 OMVs**							
D0CCD3	Uncharacterized protein	4	Unknown	0.6083	2.846	184	384	48
D0C807	Glutamate-aspartate periplasmic-binding protein	1	Periplasmic space					
D0C7Q4	Uncharacterized protein	1	Cytoplasmic membrane					
D0CG85	Elongation factor Tu (Fragment)	0						
D0CDQ1	Efflux pump membrane transporter	0						
D0C629	Translocation and assembly module subunit TamA	0						
D0CEK8	ATP synthase subunit b	0						
II.	**Unique proteins in IB010 OMVs**							
D0CF71	Carbapenem susceptibility porin CarO	6	Outer membrane	0.758	2.868	123	299	41

D0CDB4	DUF306 domain-containing protein	5	Unknown	0.570	3.053	43	140	31
D0CBN6	Uncharacterized protein	4	Outer membrane	0.624	2.898	128	249	51
D0C7A8	Lipoprotein	4	Unknown	0.664	2.993	46	120	38
D0CDQ8	Outer-membrane lipoprotein carrier protein	3	Periplasmic space					
D0C9M0	Outer-membrane lipoprotein LolB	3	Unknown	0.41	2.981	51	170	30
D0CC74	30S ribosomal protein S2	2	Cytoplasm					
D0CFX6	Metallo-beta-lactamase domain protein	2	Unknown	0.466	2.948	97	263	36
D0CDY8	TonB-dependent siderophore receptor	0						
D0CF26	Uncharacterized protein	2	Unknown	0.1651	3.176	79	84	94
D0C7G2	Uncharacterized protein	2	Unknown	0.4891	2.98	37	102	36
D0CB14	Uncharacterized protein	1	Cytoplasmic membrane					

a) B-cell epitopes predicted by Bepipred 3.0 with a threshold > 0.15; b) Subcellular location predicted with PSORTb; c) VaxiJen treshold > 0.5 d) β-barrel conformation threshold < 2.95; e) Discontinuous epitopes predicted with SEMA with a treshold > 0.51; f) 7 or more consecutive amino acid residues predicted as discontinuous epitope regions by SEMA

Table S3. Common proteins in OMVs from ATCC 19606 and IB010 with no differential expression

Protein ID	Gene	Protein Description	Combined Total Peptides
D0CAF5	HMPREF0010_01735	Peptidase, S41 family	24
OMP38	omp38	Outer membrane protein Omp38	23
D0CC38	HMPREF0010_02318	Beta-lactamase	17
D0CBL3	tolB	Tol-Pal system protein TolB	16
D0C6H3	yaeT	Outer membrane protein assembly factor BamA	13
D0C9M4	yfiO	Outer membrane protein assembly factor BamD	13
D0CC53	HMPREF0010_02333	Secretory lipase	13
D0CEG5	HMPREF0010_03145	Peptidase, M48 family	11
D0CAE4	yfgL	Outer membrane protein assembly factor BamB	10
D0CDX7	HMPREF0010_02957	Phospholipid-binding domain protein	10
D0CBL2	pal	Peptidoglycan-associated lipoprotein	9
D0CDQ0	oprM	Outer membrane efflux protein OprM	9
D0C7T2	surA	Chaperone SurA	8
D0CCW6	HMPREF0010_02596	Signal peptide protein	8
D0CD51	HMPREF0010_02681	Transglycosylase SLT domain protein	8
D0C6S2	HMPREF0010_00452	DUF6438 domain-containing protein	7

D0CCT4	sodC	Superoxide dismutase [Cu-Zn]	7
D0CE32	HMPREF0010_03012	Curli production assembly/transport component CsgG	7
D0CEW2	HMPREF0010_03292	Peptidyl-prolyl cis-trans isomerase	7
D0C5T2	csuC	Protein CsuC	6
D0C837	HMPREF0010_00917	Tetratricopeptide repeat family protein	6
D0CAH3	HMPREF0010_01753	TonB-dependent siderophore receptor	6
D0C5Z9	pgaA	Poly-beta-1,6 N-acetyl-D-glucosamine export porin PgaA	5
D0C833	HMPREF0010_00913	L-asparaginase, type II	5
D0CDG8	HMPREF0010_02798	Thiol:disulfide interchange protein	5
D0CFY3	HMPREF0010_03663	Lytic murein transglycosylase	5
D0C8R4	HMPREF0010_01144	Lipoprotein	4
D0C9H1	mtnN	MTA/SAH nucleosidase	4
D0C9S5	bamE	Outer membrane protein assembly factor BamE	4
D0CBY8	dacF	serine-type D-Ala-D-Ala carboxypeptidase	4
D0CC33	HMPREF0010_02313	ErfK/YbiS/YcfS/YnhG	4
D0CDI5	HMPREF0010_02815	Entericidin EcnA/B family protein	4
D0C8U7	HMPREF0010_01177	Peptidase M15A C-terminal domain-containing protein	3
D0CA36	mro	Aldose 1-epimerase	3
D0CAE9	pilF	Type IV pilus biogenesis/stability protein PilW	3
D0CAP1	HMPREF0010_01821	Lipoprotein	3

D0CBA6	HMPREF0010_02036	Transglycosylase SLT domain protein	3
D0CCP5	HMPREF0010_02525	LemA family protein	3
D0CF21	HMPREF0010_03351	Thiol:disulfide interchange protein	3
D0CG24	HMPREF0010_03704	OmpA family protein	3
D0C7W4	HMPREF0010_00844	Penicillin-binding protein, transpeptidase domain protein	2
D0CF50	HMPREF0010_03516	Uncharacterized protein	8
D0CBD0	HMPREF0010_02060	Signal peptide protein	6
D0CBN2	HMPREF0010_02162	Signal peptide protein	6
D0CEI7	HMPREF0010_03167	Uncharacterized protein	5
D0C626	HMPREF0010_00206	Uncharacterized protein	4
D0CCS8	HMPREF0010_02558	Signal peptide protein	4
D0CCV2	HMPREF0010_02582	Signal peptide protein	4
D0CE93	HMPREF0010_03073	Signal peptide protein	4
D0CDD2	HMPREF0010_02762	Signal peptide protein	3
D0C7G6	HMPREF0010_00696	Uncharacterized protein	2
D0C8R3	HMPREF0010_01143	Uncharacterized protein	2

Figure S1. 3D conformational epitopes predicted by SEMA.

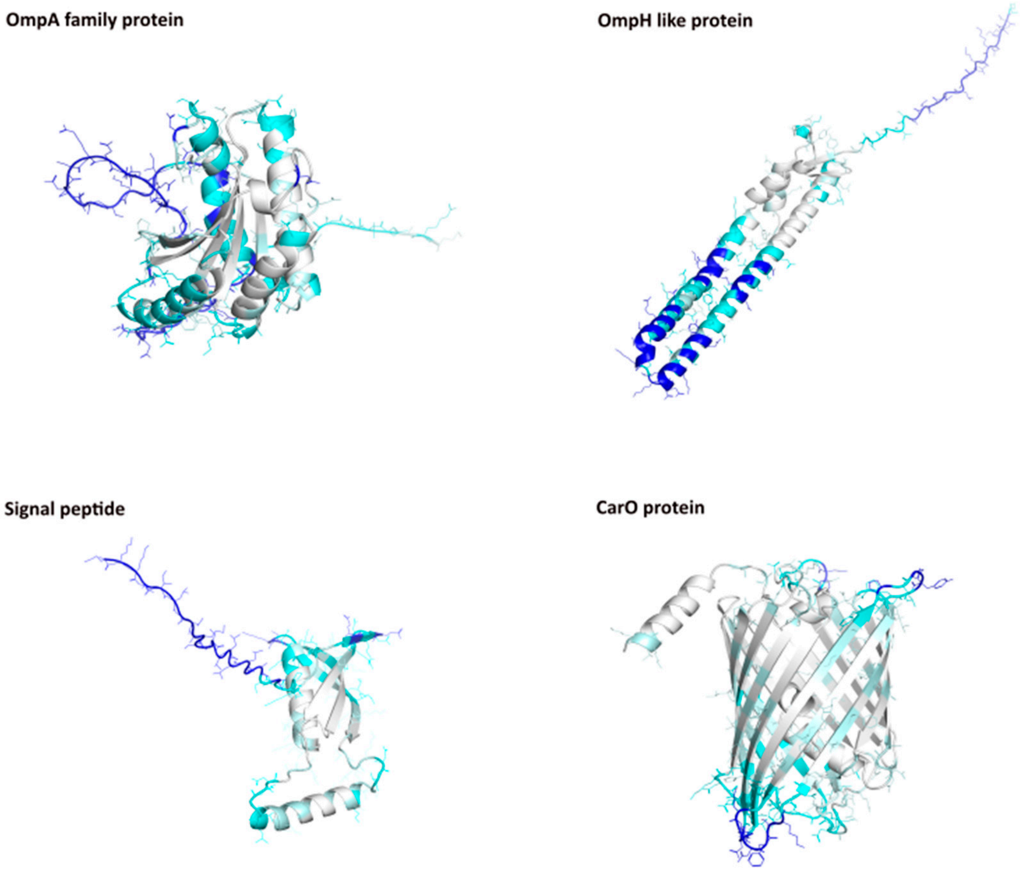

Blue and cyan regions correspond to epitopes-rich areas, while white regions represent areas not predicted to interact with antibodies (coloured in blue, SEMA score >1.1; coloured in cyan SEMA score = 0.8 – 1.1)
